# Pancreatic Ductal Adenocarcinoma (PDAC) Organoids: The Shining Light at the End of the Tunnel for Drug Response Prediction and Personalized Medicine

**DOI:** 10.3390/cancers12102750

**Published:** 2020-09-24

**Authors:** Pierre-Olivier Frappart, Thomas G. Hofmann

**Affiliations:** Institute of Toxicology, University Medical Center of the Johannes Gutenberg University Mainz, 55131 Mainz, Germany; thomas.hofmann@uni-mainz.de

**Keywords:** PDAC, 3D cell culture, organoids, personalized medicine, chemotherapy, drug response

## Abstract

**Simple Summary:**

Pancreatic ductal adenocarcinoma (PDAC) causes massive medical problems because of late diagnosis and limited responsiveness to standard chemotherapeutic treatments. This makes PDAC one of the major causes of death by cancer. To address this problem, novel tools for early diagnosis and therapy are needed. The recent development of PDAC organoids, which represent micro-scale mini-tumors, offers promising new options for personalized drug-testing based on primary PDAC patient material. This overview article summarizes and discusses the current state-of-the-art in exploiting the organoid technology to improve clinical management of PDAC.

**Abstract:**

Pancreatic ductal adenocarcinoma (PDAC) represents 90% of pancreatic malignancies. In contrast to many other tumor entities, the prognosis of PDAC has not significantly improved during the past thirty years. Patients are often diagnosed too late, leading to an overall five-year survival rate below 10%. More dramatically, PDAC cases are on the rise and it is expected to become the second leading cause of death by cancer in western countries by 2030. Currently, the use of gemcitabine/nab-paclitaxel or FOLFIRINOX remains the standard chemotherapy treatment but still with limited efficiency. There is an urgent need for the development of early diagnostic and therapeutic tools. To this point, in the past 5 years, organoid technology has emerged as a revolution in the field of PDAC personalized medicine. Here, we are reviewing and discussing the current technical and scientific knowledge on PDAC organoids, their future perspectives, and how they can represent a game change in the fight against PDAC by improving both diagnosis and treatment options.

## 1. PDAC Development and Classification

Pancreatic ductal adenocarcinoma (PDAC) is the most frequent form of pancreatic cancer with an incidence in constant progression that will represent the second cause of cancer related death in western countries by 2030 [1,2,3]. Two main models were proposed to describe the development of PDAC. In the “classical” one, acinar to ductal metaplasia (ADM) is followed by acinar-to-ductal reprogramming (ADR) and the progressive development of pre-neoplastic lesions (PanIN, pancreatic intraepithelial neoplasia) evolving towards PDAC [4,5]. The “alternative” pathway takes its origin in ductal cells that, through mutations of oncogenes such as *GNAS* but also *KRAS* [6,7,8], or tumor suppressors such as *TP53* and *RNF43* [9], form cystic lesions secreting mucin, with intraductal papillary mucinous neoplasia (IPMN) or mucinous cystic neoplasia (MCN) being the most implicated in progression to PDAC [10,11,12,13]. In PDAC, several driver genes such as *KRAS* (found in up to 90% of the cases), *TP53*, *CDKN2A*, or *SMAD4* were identified together with many additional mutations, generating an extremely high intra- and inter-tumoral heterogeneity [14,15,16]. Several studies have attempted to classify PDAC in several subtypes. Gene expression-based profile studies using mainly resectable tumors, stratified PDAC into 3 (the “*classical*”, the “*quasimesenchymal* (QM)”, and the “*exocrine-like*”) [14] or 4 subtypes (the “squamous”, the “ADEX” (abnormally differentiated endocrine, exocrine), the “pancreatic progenitor”, and the “immunogenic”) [17]. Two additional studies considering the microenvironment classified PDAC into 2 (“*basal-like*” and “*classical*”) [18], or 5 subtypes (“*pure classic*”, “*immune classic*”, “*pure basal like*”, “*stroma activated*”, and “*desmoplastic*”) [19]. Waddell et al., using whole genome sequencing (WGS), identified 4 groups based mainly on their variation on chromosomal structure: The “*stable*”, the “*scattered*”, the “*unstable*”, and the “*locally arranged*” [20]. All these different subtypes are proposed to be associated with a prognostic outcome and differential drug responsiveness. More recent works were able to classify PDAC based on their epigenetic landscapes [21], and the analysis of a cohort of primary tumors and metastases from 314 patients by WGS and whole transcriptome sequencing (WTS) observed 4 signatures that summarized in some ways all the former published classifications (“*basal-like-A*”, “*basal-like-B*”, “*hybrid*”, “*classical-A*”, and “*classical-B*”) and demonstrated, using single-cell analysis, that various subtypes can coexist within one tumor [22], indicating a high genetic heterogeneity in PDAC.

## 2. The Challenges of Pancreatic Ductal Adenocarcinoma (PDAC)

However, despite intensive efforts to provide oncogene and tumor suppressor mutational and transcriptional maps, PDAC prognosis has not been significantly improved for the past 30 years with 5-year survival still below 10% [23,24]. The bad prognosis is mainly due to late diagnosis, high genetic heterogeneity, lack of efficient treatment, and limited surgical procedures. Indeed, most of the patients are diagnosed at an advanced stage when metastases have already disseminated and consequently less than 20% of the patients are benefiting from tumor resection. Of note, despite providing a significant gain of 5-year survival, the surgical procedure still remains a challenging operation with high operative and postoperative risks [25,26]. In addition, a post-operation PDAC recurrence is observed in up to 80% of the cases [27,28,29]. Currently, chemotherapies using gemcitabine/nab-paclitaxel [30,31] or FOLFIRINOX (5-Fluoruracil, leucovorin, irinotecan, and oxaliplatin) [32,33] remain the gold standard for PDAC treatment with a significant average increase of survival of up to 1 year.

Therefore, the identification of biomarkers allowing early detection of PDAC or the development of tools to promote efficient drug guidance in the frame of personalized medicine is in critical need. One of the first attempts in this direction was the establishment of adherent PDAC cell lines (2D). However, 2D cells are shown to diverge significantly from the initial tumor by many aspects including polarity, microenvironment, cell metabolism, and gene expression. In addition, the relative low take rate and their lack of drug response-predicting value precluded their use in clinical routine [34]. The next step on the road towards personalized medicine was patient derived xenografts (PDX) which, in contrast to 2D, recapitulate more closely the primary tumor and allow prediction of drug responses. To this point, PDX were generated with a heterogenous success rate from PDAC primary tumors, fine needle aspiration (FNA), and metastases [35,36,37,38,39,40,41,42]. Although the PDX revealed itself as a very interesting and promising tool in other tumor entities, due to time constrains of PDAC management and personalized medicine, it is too slow. Therefore, three-dimensional (3D) cell culture represented by organoids that recapitulates more closely the primary tumor without the disadvantages of PDX opened entirely new perspectives within the PDAC field [43]. Indeed, 3D organoids reproduce largely the morphology, gene and protein expression, cell polarity, and cellular metabolic heterogeneity of the primary tumor, and they appear to be genetically stable over serial passages, which makes them an ideal tool for PDAC modeling and drug testing [44,45,46].

## 3. PDAC Organoids

### 3.1. What Is an Organoid?

**Figure 1:**
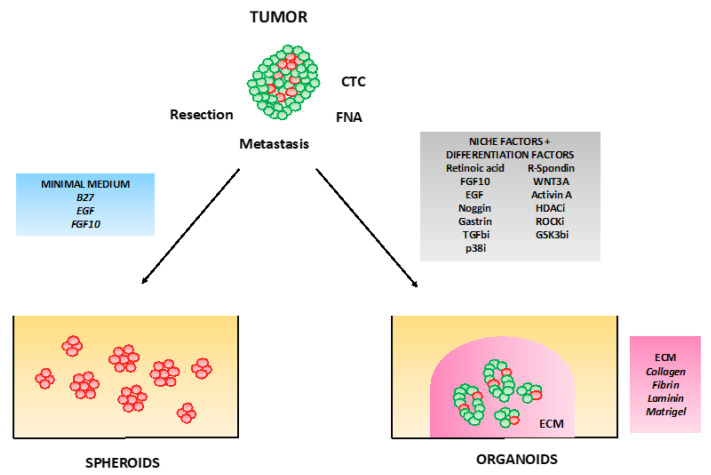
Spheroids vs. organoids. Spheroids or spheres are obtained by tissue or cancer stem cells growth in a minimum serum-free medium. Their spherical morphology triggers an oxygen and nutrient gradient towards the center, leading often to massive cell death in the center of the structure. Spheroids are lacking the capacity to form tissue-like structure. Organoids in contrast to spheroids require stem cell niche factors and extra-cellular matrix (ECM), which allow stem cells to express their differentiation and self-organization capacity. In addition, this structure due to the presence of a lumen does not exhibit a hypoxic or nutrient gradient. CTC: Circulating tumor cells, FNA: Fine needle aspiration. Red cells: Stem cells, green cells: differentiated cells.

The term organoid (organ-like) is not new, since it was used for the first time in 1946 [47]. However, by the establishment of intestine organoids by Sato et al. in 2009 [48], the field has expanded exponentially year by year. It is now possible to isolate primary and tumor organoids from most tissues including breast, pancreas, and liver [49]. However, there are still recurrent discussions regarding what should be and should not be named an organoid. The confusion surrounding this term is obviously fueled by the close proximity of the organoid and spheroid fields and by the recurrent misusage of these terms as synonyms. Furthermore, the development of a more and more sophisticated spheroid culture system with molecular, cellular, and architectural characteristics approaching those of organoids additionally increased the complexity (Figure 1). Of note, the three-dimensional culture field encompasses two main domains: Spheroids (multicellular tumor spheroids (MCTS), tumorspheres, tissue-derived tumor spheres (TDTS), organotypic multicellular spheroids (OMS)) and organoids (review in [50,51,52]). Several definitions for organoids were proposed (review in [53]). The current consensus seems to build around four criteria to define a genuine organoid [54]. An organoid is a 3D cellular structure: (1) It recapitulates the identity of the organ it is supposed to model, (2) it mirrors the organ’s cell type diversity, (3) it reproduces the organ specific functions, and (4) it follows the same self-organization of the tissue it should reproduce. The cells of origin can be a primary or tumor tissue, embryonic stem (ES) or induced pluripotent stem (iPS) cells (Figure 1).

### 3.2. Establishment of Mouse and Human PDAC Organoids

In the pancreas, the isolation and establishment of organoids were first performed in mice [55,56] and progressively, multiple methods were developed in humans to isolate and culture PDAC or primary ductal organoids to ensure their long-term culture and cryopreservation [45,57,58,59,60,61,62,63,64]. All these methods (Table 1) can be classified based on their fetal bovine serum (FBS) content: FBS high [63,65], FBS-reduced [41,45,55,57,59], and FBS-free [41,56,60,62,64]; or on the type of scaffold used to culture the organoids: Collagen [56], solubilized basement membrane preparation extracted from the Engelbreth–Holm–Swarm mouse sarcoma (BME2, Matrigel) [41,45,55,57,59,60,62,63,64], and air–liquid interface [65,66].

Organoids are usually obtained by an initial mechanical dissociation of the tissue, then an enzymatic digestion with collagenase/dispase followed by TrypLE or accutase treatment. The success rate seems to highly depend on the quantitative and qualitative characteristics of the sample and the use of the Rho kinase inhibitor (Y267632) in all the steps of the isolation procedure and for the first passage. Notably, several well-detailed protocols for mouse or human organoid generation developed by the Tuveson lab [67,68] (http://tuvesonlab.labsites.cshl.edu/protocolsreagents) and the Muthuswamy lab (https://www.muthuswamylab.org) are freely available.

Currently, two methodological philosophies prevail in the pancreas organoid field defined by the addition or not of WNT to the medium. The Tuveson group and others favor the use of WNT into organoid medium, while the Muthuswamy lab elaborated using their intensive work on hES differentiation into pancreas progenitors, a chemically defined WNT-free tumor organoid medium [60]. Along the same line, the Sato group showed that, in contrast to a previous study [57], a portion of PDAC organoids were independent of both exogenous WNT and R-SPONDIN and that human PDAC segregate into three main subtypes (i) WNT- and R-SPONDIN-dependent (W-), (ii) WNT-independent/R-SPONDIN-dependent (W+), (iii) WNT- and R-SPONDIN-independent (WRi), based on their exogenous WNT niches dependency [62]. These three subtypes recapitulate the different stages of PDAC development from PanIN to metastasis. These data strongly suggest that the lack of WNT/R-SPONDIN selects for the most aggressive form of PDAC during the establishment steps and that the use of one medium or the other preselects certain subtypes of PDAC organoid.

### 3.3. The “PDACoids”

**Figure 2:**
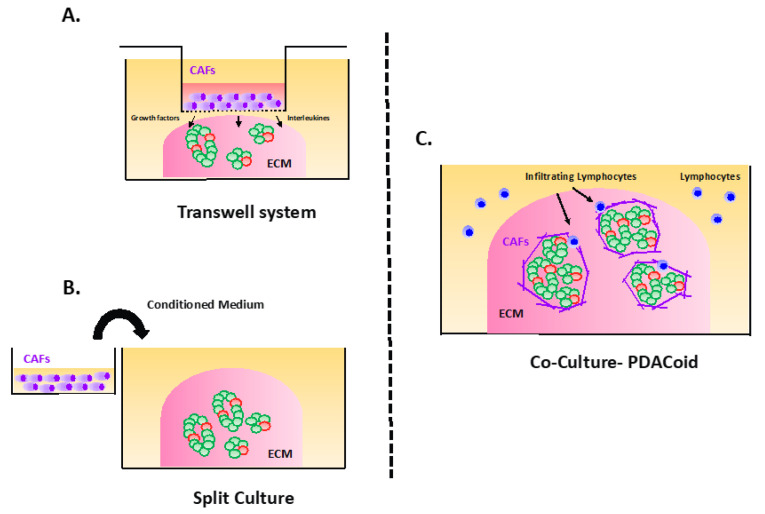
Pancreatic ductal adenocarcinoma (PDAC) organoids co-culture with cancer-associated fibroblasts (CAFS) and immune cells: “the PDACoids”. (**A**) Using conditioned medium, (**B**) using Transwell^®^ system, and (**C**) “*three-dimensional PDACoids*”.

PDAC is composed of two entities: The tumor cells themselves and a dense desmoplastic reaction also known as the stroma, composed of cancer-associated fibroblasts (CAFs), extra-cellular matrix (ECM), pancreatic stellate cells (PSCs), endothelial cells immune cells, and various growth factors. The stroma represents up to 90% of the tumor volume and forms a physical barrier preventing drug delivery contributing to drug resistance. It is also essential for PDAC growth by providing ECM components, cytokines, and growth factors, amongst others. Therefore, it seems that all the components of the stroma are required in the tumor organoid culture to closely model PDAC ex vivo. Several groups developed 3D-coculture with organoids, fibroblasts and immune cells. We decided to arbitrarily name this complex structure: “*PDACoids*” (PDAC-like), to discriminate them from the conventional tumor organoids which are cultured without fibroblasts and immune cells (Figure 2).

Of note, the incorporation of fibroblasts or immune cells into the culture often requires the removal of several components that inhibit the growth of fibroblasts (Noggin, B27, and TGFb inhibitor) and supplement with a small proportion of FBS. Notably, Öhlund et al. revealed that distinct sub-populations of inflammatory fibroblasts and myofibroblasts were able to propagate “*2-dimensional-PDACoids*” (tumor organoid and CAFS) for several passages in a minimum medium composed only of DMEM and 5% FBS. The presence of CAFs was shown to be beneficial for the organoid growth, even in the absence of essential components in the organoid medium. Another study reached similar conclusions this time using human PDACoids [62]. Interestingly, it was also shown that a bioprinting of a scaffold can support “2-dimensional-human PDACoids” [69]. Finally, a more complex and elaborated approach was achieved by Tsai et al., who cultured in organoid medium [57] “3-dimensional PDACoids”, including immune cells that exhibited a significantly lower sensitivity to chemotherapies compared to the tumor organoids without immune cells [61]. Therefore, PDACoids appear to reproduce more closely PDAC and may represent a superior model for drug testing. However, it is currently not clear how this complex technology could be implemented in daily clinical routine work.

### 3.4. Towards a Chemically Defined PDAC Organoid Culture

Despite all the improvements, several aspects of organoid culture are still susceptible to high variation and therefore have negative inputs on data reproducibility that could limit their clinical application. First, the preparation of WNT-conditioned medium, which is still one of the key components of the majority of PDAC organoid culture media, relies on mouse cells overexpressing and secreting WNT into the medium. In addition, the secretion and stabilization of active WNT due to its its physical properties in cultured cells requires bovine serum [70]. The batch-to-batch variation is high, and obtaining a standard WNT-conditioned medium is cumbersome. It could explain why some groups are favoring commercially prepared medium to reduce this variation as much as possible [45,61]. Of note, the activity of WNT is analyzed by indirect measurement methods such as the TOP/FOP-Flash assay [71,72,73], reporter cell lines (review in [74]), or immuno-detection of phosphorylated proteins [75,76]. Surprisingly, the TOP/FOP ratio, which is a key parameter to evaluate the quality of the batch preparation, is often excluded in the vast majority of publications on organoids. To overcome this conditioned medium hurdle, the elaboration of a WNT/R-SPONDIN1-based chemically-defined medium has been a key objective for a long time. While recombinant R-SPONDIN1 or NOGGIN suitable for organoids culture are easily available either from commercial providers or by homemade alternatives [77,78], the production and purification and long-term conservation of recombinant, active stable WNT remains a technical challenge (https:web.stanford.edu/group/nusselab/cgi-bin/wnt/purification) [79]. Notably, several recombinant WNT ligands are commercially available, but were not shown yet to be used for PDAC organoid culture. Nonetheless, the recent discovery by Mihara et al. that the glycoprotein AFAMINE forms a complex with WNT3A and other WNT subtypes and promotes their solubility and sustains their activity in aqueous buffer [80] opened the door to the production of FBS-free WNT-conditioned medium and purification of stable, active WNT3A. This work was complemented by the work of Tuysuz et al., describing that WNT3A combined with soluble lipid carriers including liposomes and hydrophobic nanoparticles (NPs) exhibited enhanced activity [81].

ECM-derived from Engelbreth–Holm–Swarm mouse sarcoma (Matrigel, BME) became the standard scaffold for pancreatic 3D organoid culture mainly due to its quality to support organoid growth and its convenience of use compared to other products such as collagen. However, its murine cancer origin and not well-defined growth factor composition, along with its batch-to-batch variation, are raising more and more questions in the frame of its clinical use. Several approaches involving hydrogels of natural or synthetic polymers, “organ on chip”/bioprinting, and native ECM derived from organ decellularization have been developed to grow organoids from various tissues [82,83,84,85,86]. Surprisingly, only few studies reported the development of Matrigel alternatives specifically for pancreas or PDAC organoids. A first study showed that pancreas progenitor organoids derived from differentiation of hES cells could be maintained in a synthetic soft PEG-hydrogel that was covalently functionalized with laminin 1 [87]. A second study described the preparation of a native ECM scaffold and hydrogel from human pancreas [88]. Though this second method definitely offers a better scaffold by reproducing more closely a human pancreas microenvironment, it clearly does not solve the criticism on the batch-to-batch variation, and has not yet shown its efficiency on sustaining growth of tumor organoids. A third study, which seems to be the most promising achievement on the way to replace Matrigel, reported a well-defined fibrin–laminin hydrogel able to efficiently support the growth of various organoids including PDAC and pancreas organoids [89]. Finally, Hou et al. demonstrated that they were able to grow primary PDAC organoids together with their respective cancer-associated fibroblasts (CAFs) using bioprinting technology [69]. Altogether, despite of intensive research and technical development, Matrigel remains the standard scaffold for organoid culture.

## 4. PDAC Organoids and Personalized Medicine

### 4.1. Sources of PDAC Organoids

The organoid technology allows for an efficient and reliable isolation of PDAC cells from various sources such as resected tumors, FNA, and metastases. A success rate of between 62% to 100% for resected tumor and FNA, and up to 70% for PDAC liver metastases is reported [57,60,61,63,78,90,91]. These results outperformed by far those obtained with 2D culture and PDX and represent a significant improvement in PDAC management and in personalized medicine, since it is now possible to rapidly obtain biological materials in sufficient quantity and quality from almost all PDAC patients.

### 4.2. CTC-Derived Organoids

A fourth source of tumor material has just been starting to be exploited in pancreas: The circulating tumor cells (CTCs) and their “non-tumorigenic” counterparts, the circulating epithelial cells (CECs) [92,93,94,95]. CTCs represent a promising and nearly unlimited tissue source alternative to invasive biopsies even if they are very rare in blood (1 CTC per 10^7^ to 10^9^ blood cells). Furthermore, CTC number and dynamics were shown to correlate with tumor progression and the response to treatment. Notably, new alterations in CTCs were even identified long before disease recurrence, indicating that CTC can be used as a predictive marker to anticipate the relapse and establish new therapeutic strategies. CTCs, despite their relative rarity, were isolated in blood from PDAC patients (review in Martini et al., 2019) prior and even after therapy using various standard methods such as CellSearch^®^ [96], ScreenCell^®^ system [97,98,99,100], anti-cytokeratin/anti-EpCam MACS enrichment [101], isolation by size of epithelial tumor (ISET) cells assay [102], and microfluid platform [103]. In PDAC patients, CTCs were shown to be heterogenous [104]. A first study identified two CTC subtypes: The “*epithelial*” CTCs (eCTCs) and the “*epithelial/mesenchymal*” CTCs (mCTCs) [102]. A recent study classified PDAC CTCs in 3 categories “epithelial”, “mesenchymal”, and “hybrid”, based on E-cadherin and vimentin expression [105]. CTCs travel in clusters that retain cell–cell contacts. Moreover, mesenchymal features have been observed in CTCs isolated from blood of metastatic cancer patients and these CTCs seem to display a higher metastatic potential than single migratory cells [106]. Remarkably, CTCs have also been shown to cluster with multiple immune cell types [107]. Finally, CECs were also isolated from patients with cystic lesions such as intraductal papillary mucinous neoplasia (IPMN), mucinous cystic neoplasm (MCN), and other pancreas diseases such as pancreatitis [108,109,110]. Even if the scientific literature on the establishment of organoids from CTCs is still limited (review in [111]), two successful attempts were described. Gao et al. demonstrated that organoids can be established from prostate cancer CTCs [112], and Zhang et al. were able to establish lung cancer organoids using a 3D-co-culture system [113]. In pancreas, CTCs together with immune cells embedded in Matrigel were able to be co-cultured for up to 7 days [107]. Altogether, these data indicate that it is possible to isolate CTCs from PDAC. Nevertheless, further investigations are required to reliably establish organoids from CTCs and perform drug guidance.

### 4.3. Drug Guidance Based on Patient-Derived Organoids (PDO)

The feasibility and reliability of drug testing based on organoid cultures was first shown elegantly in gastrointestinal cancers by demonstrating that PDO exhibited 100% sensitivity, 93% specificity, 88% positive predictive value, and 100% negative predictive value for the drug response [114]. Following this example, several groups took the path to validate PDOs as a drug response predicting tool in PDAC [41,45,78,91,115,116,117]. The most advanced studies involving numerous PDO cell lines and patient follow-ups demonstrated a high correlation between the PDAC PDO response and the clinical patient’s response for the gemcitabine–paclitaxel and FOLFIRINOX treatments [78,91]. Tiriac et al. combined the transcriptional profile and pharmacotyping results of PDO to define a pharmacotranscriptomic signature for the drug response to gemcitabine, paclitaxel, and oxaliplatin [91]. In addition, they compared the drug response and genomic/transcriptomic signatures between PDO derived from ascites, liver, and diaphragm metastasis of the same patient. Interestingly, they observed minor variations on copy-number alterations and mRNA expression between the cell lines but a heterogeneous drug response. Taken together, these findings suggest the co-existence of multiple cancer sub-clones in metastatic patients defining differential drug responses. This needs to be considered for drug guiding and could be one of the explanations for partial/limited responses observed in some of the patients. One remaining question is how the components of the medium affect the molecular signature of the organoids and the drug response profile. It was shown that removal or addition of specific components, such as EGF, selects for some mutations in organoids and prevents overgrowth of contaminating wild-type cells [62]. A recent study based on this finding compared paired sets of PDO cultured with or without WNT, Activin receptor-like kinase inhibitor (A83-01) and noggin and demonstrated that the resulting PDO cultures were genetically different reproducing the intra-PDAC heterogeneity [78]. These data were corroborated by the small proportion (38%) of paired PDO cell lines being able to grow in both media. Unfortunately, no comparison of drug response profiles was performed, thus postponing the answer to the central question of which medium provides the best predictive value. Finally, both studies validated that PDOs have an equivalent efficiency of prediction for targeted therapies such as Olaparib, MEK, and PRMT5 inhibitors compared to genetic-based targeted medicine [62,78]. Finally, organoids were shown to efficiently generate tumors that recapitulate the parental tumor in subcutaneous or orthotopic xeno- and allograft models [41,57,60,118]. As few as 50,000 cells per injection were sufficient to trigger the formation of a PDAC in 4 to 7 weeks [60,118]. In addition, in contrast to 2D cells that quickly form PDAC, primary tumor organoids generate first PanIN-like lesions that progress very slowly to full blown PDAC [57,118]. Recently, it was shown that transplantation of PDAC organoids directly into murine pancreatic ducts allows reproducing the different subtypes coexisting in one PDAC [118]. Of note, Boj et al. reported that mouse PDAC organoids could be successfully transplanted in immune-proficient C57BL/6 as well as in nude mice [57].

Technically, organoid drug testing is performed in various formats: 96-well, 384-well, and even 1536-well plate. Usually, the cells are seeded on Matrigel-coated plates and cultured with organoid medium containing 5% to 10% of Matrigel [60,78,91,119]. Recently, for the first time, a bioprinting engineered plate was used for a high throughput drug screen [69], which allowed for a significant downsizing and increase in the number of drugs tested. Most of the studies are using the luminescence ATP-based assay CellTiter-Glo^®^ 3D cell viability assay but ATP-independent assays such as Cytotox-Glo™ Cytotoxicity and CyQUANT™ assays [60] are also used and provide reproducible results. Recently, several straight forward cost-effective methods including the FACS-based fluorometric method [120] and bright-field imaging [121] were shown to exhibit comparable sensitivity to the CellTiter-Glo^®^ 3D cell viability assay. The drug testing is performed by dose response followed by area under the curve (AUC) measurement, which provides the best correlation with patient response [122]. One major discrepancy, which is rarely mentioned between all the studies, is the time interval between cell seeding and the start of the treatment which differed between 1 to 4 days. Surprisingly, until now, no study in the pancreas analyzing whether organoid size or proliferation status may influence the drug response profile, or which time point/size gives the highest prediction rate has been reported to date.

## 5. Conclusions and Perspectives

**Figure 3:**
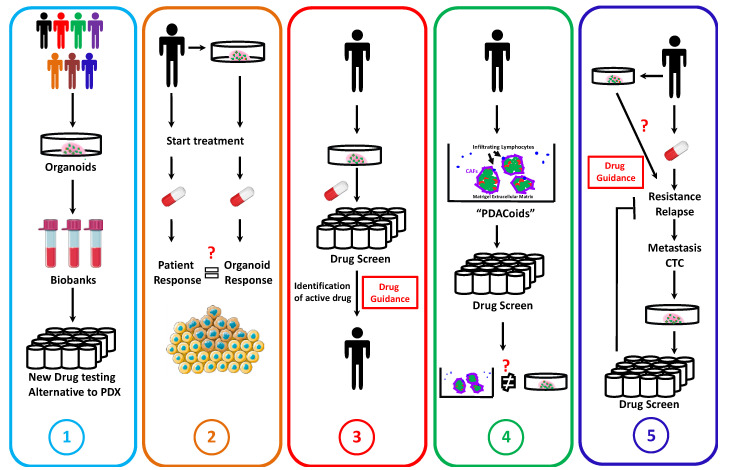
Current and future applications of PDAC organoids in personalized medicine. (**1**) Creation of biobanks of PDAC organoid cell lines allowing for the validation of new treatments during pre-clinical studies. (**2**) Validation of organoids as drug predicting tool. (**3**) “Real-time” drug guidance for PDAC patients using organoids. Isolation of organoids and drug test will allow us to determine the most efficient therapy against the patient PDAC. (**4**) Development of “*PDACoids*” to improve the drug sensitivity prediction and to test whether they are providing a better prediction than conventional tumor organoids. (**5**) Strategy involving organoids to counteract drug resistance acquisition and to propose alternative therapeutic strategies. After the relapse, if possible, a second metastasis biopsy or the isolation of circulating tumor cells will allow for the isolation of resistant organoids and determine an alternative to the former therapy. Adapted from [49]. Creative Commons Attribution 3.0 Unported License Servier Medical Art by Servier.

In the past few years, several studies provided significant contributions to develop methods for PDAC organoid cultures, to create biobanks, and to validate their use for drug screening (Figure 3).

All these studies demonstrated that organoids recapitulate the morphology and molecular signature of the primary tumor and are useful to predict drug responses. Standing alone, this represents a tremendous game change in PDAC-handling options since, for the first time, a tool allowing faithful drug sensitivity prediction in real-time is available.

**Figure 4:**
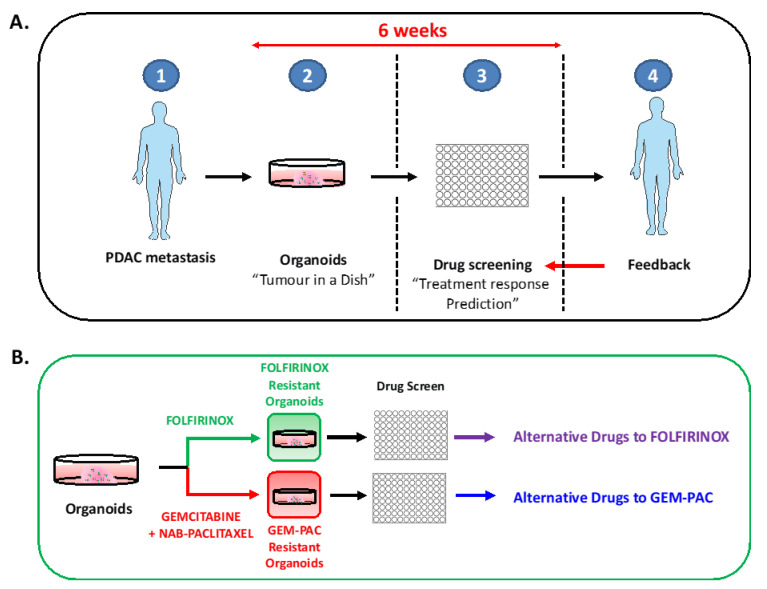
Scheme of “real-time” drug testing in metastatic PDAC and anticipation of drug resistance. (**A**) Organoids are isolated from liver metastasis (1–2) and expanded for 4 to 6 weeks prior performing the drug screen (3). The result is compared with the response of the patient and the treatment can be modified according to the drug sensitivity profile (4). (**B**) Anticipation of the resistance to the first-line therapy: Gemcitabine–paclitaxel and FOLFIRINOX using organoids. Creative Commons Attribution 3.0 Unported License Servier Medical Art by Servier.

Nevertheless, additional work is required to demonstrate that this prediction could be reliably applied to metastatic PDAC patients where time is short due to fast disease progression (Figure 4A). Notably, resected tumor-derived organoids can be established in two to three weeks and the drug screen can be performed in 4 weeks, while metastasis-derived organoid establishment and drug screen often require more than 6 weeks. Therefore, patients with metastatic cancer, when it is possible, will receive a first-line therapy long before a PDO-based drug sensitivity profile is available. Currently, no studies in the pancreas have reported how this initial treatment is affecting the results of the drug screen. Of note, two recent studies indicated that upon chemotherapy administration, the PDAC subtype can shift, but no correlation with drug response has been performed [22,123]. One suitable solution could be to incorporate an additional step in the drug test pipeline, triggering resistance to the first line therapy before the first drug screen (Figure 4B). This strategy could eventually be used in patient management to predict the acquisition of drug resistance and adjust the treatment accordingly. In addition, in the frame of clinical applications, significant standardization efforts have to be performed. Indeed, even if there is a consensus on two types of media WNT-dependent and WNT-independent that select for different sub-clones of the tumor, practically every single research group has developed its own isolation, culture, and drug response analysis protocols, which hampers a comparison between those studies. Moreover, elaborated studies defining whether and how these media trigger a different drug sensitivity are required.

Finally, the successful work of several groups in differentiating hES cells and iPSCs into pancreas progenitors [60,124,125,126,127,128], and in the near future into ductal and acinar cells, opened completely new perspectives in modeling PDAC. Indeed, it is now possible to use iPSCs from PDAC-predisposed patients or to engineer specific PDAC-associated mutations directly in hES with CRISPR/CAS9 in order to mimic, monitor, and visualize in vitro the different stages of PDAC development and to identify key biological markers (i.e., metabolism, cell markers, microenvironment), allowing for an earlier detection. Hypothetically, this approach could ultimately allow for the prediction of risk factors, genetic signatures, and drug responses of PDAC even prior to the occurrence of the tumor itself.

Collectively, the development of PDAC organoids and their clinical implementation has opened a new era in PDAC therapy. Indeed, even though the expectations are high, the first pre-clinical and clinical experimentations seem to justify all the hopes placed in them. However, like many scientific studies before, it is crucial to establish robust standards to pave the road for the routine use of this magnificent tool.

## Figures and Tables

**Table 1:** Summary of the different methods to culture mouse and human organoids.

Reference	Medium	ECM/Matrix	Tissues
Huch et al., 2013 [55]	AdvDMEM/F12, R-spondin1-conditioned medium (10%), Noggin-conditioned medium (10%), B27 (1×), N-acetylcysteine (1.25 mM), EGF (50 ng/mL), gastrin I (10 mM), FGF10 (100 ng/mL), nicotinamide (10 mM), Y-27632 (10 µM)	Matrigel 100% (Dome)	Primary mouse tissue Mouse PDAC
Reichert et al., 2013 [56]	AdvDMEM/F12, D-glucose (4.7 mg/mL), nicotinamide (1.22 mg/mL), soybean trypsin inhibitor (0.1 mg/mL), Primocin (0.2%), Nu-Serum IV (5%), ITS + premix (0.5%), bovine pituitary extract (25 µg/mL), mEGF (20 ng/mL), cholera toxin (100 ng/mL), T3 (5 nM), Dexamethasone (5 nM)	Collagen	Primary mouse tissue Mouse PDAC
Li et al., 2014 [65]	Ham’s F12, FCS (20%)	3D collagen gel/double dish culture system/air–liquid interface	Primary mouse tissue Mouse PDAC
Boj et al., 2015 [57]	AdvDMEM/F12, HEPES (1×), WNT-conditioned medium (50%), R-spondin1-conditioned medium (10%), Noggin-conditioned medium (10%), GlutaMAX (1×), B27 (1×), N-acetylcysteine (1mM), EGF (50 ng/ mL), gastrin I (10 mM), FGF10 (100 ng/mL), nicotinamide (10 mM), A83-01 (0.5 µM), PGE2 (1 µM), Primocin (1 mg/mL)	Matrigel 100% (Dome)	Primary human tissue Human PDAC
Huang et al., 2015 [60]	AdvDMEM/F12, B27 (1×), ascorbic acid (50 µg/mL), insulin (20 µg/mL), hydroxycortisone (0.25 µg/mL), FGF2 (100 ng/mL), all-trans retinoic acid (100 nM), Y-27632 (10 µM)	Coated Matrigel 100% + Medium 5% Matrigel	Human PDAC
Walsh et al., 2016 [63]	RPMI, FCS (10%), EGF (10 ng/mL)	Matrigel 50%	Human PDAC Mouse PDAC
Broutier et al., 2016 [59]	AdvDMEM/F12, HEPES (10 mM), R-spondin1-conditioned medium (5%), GlutaMAX (1×), B27 (1×), N-acetylcysteine (1 mM), EGF (50 ng/mL), gastrin I (10 mM), FGF10 (100 ng/mL), nicotinamide (10 mM), Noggin (25 ng/mL)	BME2 100% (Dome)	Primary mouse tissue
Broutier et al., 2016 [59]	AdvDMEM/F12, HEPES (10 mM), WNT-conditioned medium (50%), R-spondin1-conditioned medium (10%), GlutaMAX (1×), B27 (1×), N-acetylcysteine (1 mM), EGF (50 ng/mL), gastrin I (10 mM), FGF10 (100 ng/mL), nicotinamide (10 mM), A83-01 (5 µM), N2 (1%), PGE2 (3 µM), Noggin (25 ng/mL)	BME2 100% (Dome)	Primary human tissue
Lee et al., 2017 [64]	AdvDMEM/F12, hEGF (50 ng/mL), hR-spondin-1 (500 ng/mL), hFGF10 (50 ng/mL), mNoggin (100 ng/mL), nicotinamide (10 mM)	GFR-Matrigel 100% (Dome)	Primary human pancreas tissue
Seino et al., 2018 [62]	AdvDMEM/F12, HEPES (10 mM), GlutaMAX (2 mM), B27 (1×), gastrin I (10 nM), N-acetylcysteine (1 mM), mEGF (50ng/mL), Noggin (100 ng/mL), R-spondin1-conditioned medium (10%), Afamin-Wnt-3A-conditioned medium (25%), A83-01 (500 nM), SB202190 (10 µM)	GFR-Matrigel 100% (Dome)	Human PDAC
Tsai et al. 2018 [61]	IntestiCult™ (mouse), B27 (1×), gastrin I (10 nM), hEGF (100 ng/mL), hFGF10 (100 ng/mL), Nicotinamide (10 mM), A83-01 (500 nM), N-acetylcysteine (1.5 mM), Primocin (1 mg/mL), Y-27632 (10.5 µM)	GFR-Matrigel 100% (Dome)	Human PDAC Primary human pancreas tissue
Romero-Calvo et al., 2018 [45]	IntestiCult™ (mouse), B27 (1×), gastrin I (10 nM), FGF10 (100 ng/mL), nicotinamide (10 mM), A83-01 (500 nM), N-acetylcysteine (10 mM), Primocin (1 mg/mL), Y-27632 (10.5 µM)	GFR-Matrigel 100% (Dome)	Human PDAC-PDX and primary tumors
Choi et al., 2019 [41]	AdvDMEM/F12, B27, N-acetylcysteine, EGF, FGF10, Rspondin-1, Noggin *	GFR-Matrigel 100% (Dome)	Human PDAC-PDX from metastatic PDAC

* The exact composition of the medium is not provided in the manuscript.
